# Challenges in the structural science of materials

**DOI:** 10.1107/S2052252516010022

**Published:** 2016-06-27

**Authors:** C. Richard A. Catlow

**Affiliations:** a Department of Chemistry, University College London, 20 Gordon St., London WC1H OAJ, UK; b School of Chemistry, Cardiff University, Cardiff CF10 3AT, UK

**Keywords:** structural science, materials, editorial

## Abstract

Articles published recently in **IUCrJ** continue to exemplify the developments and challenges in the structural science of materials.

The articles published recently in **IUCrJ** continue to exemplify the developments and challenges in the structural science of materials. They illustrate the exciting rapid developments in technique, sources, instrumentation and data analysis, the continuing role and growing power of computation in the field, and the increasing ability to investigate complex structural problems in materials that are of direct relevance to their function.

Advances in technique and data analysis are well illustrated by the review article of Van Aert *et al.* (2016 ▸) which describes how developments in transmission electron microscopy coupled with novel data analysis methods can give accurate structural information on nanostructured and disordered materials; and illustrates how increased precision can be achieved in atomic positions using these techniques. Several examples illustrate the power of the approach, for example the accurate information on atomic coordinates adjacent to a twin boundary in CaTiO_3_, as illustrated in Fig. 1 ▸.

Significant developments in techniques are also described in the article of Rius *et al.* (2015 ▸), who describe how the synchrotron-based through-the-substrate X-ray micro-diffraction technique (tts-μXRD) can now be used to obtain structural information on microvolumes of crystals embedded in a complex matrix – a development of importance for several areas of materials science including the study of complex mineralogical samples. The use of synchrotron radiation *in operando* methods in structural science, particularly relating to catalytic systems, continues to grow as illustrated by the recent study of Lezcano-González *et al.* (2016 ▸) who investigated complex structural changes during the operation of a microporous de­hydro­aromatization catalyst.

A core area of crystallography is of course the determination and modelling of electron density, recent developments in which are discussed in the article of Macchi *et al.* (2015 ▸), who emphasize multi-technique approaches, and show the importance of developments in sources and detectors and of new multipole models for describing charge densities accurately. Related topics are considered in the article of Sanjuan-Szklarz *et al.* (2016 ▸), which discusses how improved accuracy may be obtained by using transferable aspherical atom models (TAAM) in structure refinements and how such an approach can yield high-quality structures even from low-resolution data.

Several articles describe the role of PDF analyses in structural studies of disordered materials. Whitfield *et al.* (2016 ▸) examine the perennially important topic of cation displacements in perovskite structured ferroelectrics, focusing on the widely studied PZN [(Pb/Zn)NbO_3_]. They show how PDF data from powders yield valuable information on lead cation displacements, while at the same time finding that such analyses could not distinguish some of the features that could be observed in analyses of single-crystal diffuse-scattering (SCDS) data. The theme of local structures in ferroelectric perovskites obtained from PDF and SCDS analyses is further discussed in the article of Keen (2016 ▸). Jensen *et al.* (2015 ▸) explore the use of PDF approaches in the study of thin films and show how the information obtained on local structure helps open up the field of local structural analyses of thin films and gives insight into crystallization processes. While Scavini *et al.* (2015 ▸) combine PDF analyses with ESR to reveal fascinating new information on the widely studied solid electrolyte Gd/CeO_2_ and obtain unique information on both local and intermediate range structure in this disordered material by showing how small defect clusters condense into nano-domains. The role of PDF in exploring meso-scale structure is further discussed and illustrated in the article of Egami (2015 ▸).

Two articles illustrate how structural science is now able to give insight into crystal fracture and crack propagation – one of the most widely studied problems in materials science. In a pioneering study, Rack *et al.* (2016 ▸) show how high-speed crack propagation in silicon wafers can be investigated *in situ* under thermal stress and imaged simultaneously in direct transmission and diffraction X-ray imaging. By obtaining movies of the process they are able to show that tip propagation is not a continuous process, but occurs by jumps in the tip position – information which is of real value to the semiconductor industry. The topic is further discussed in the article of Tanner (2016 ▸) who comments that ‘It is quite extraordinary that over the 57 years from the invention of X-ray topography by the late Andrew Lang, exposure times have gone from the best part of a day to a microsecond’. The key is, of course, improvements in both sources and detectors.

The role of modelling in structural science is well illustrated by the article of Giberti *et al.* (2015 ▸) who describe the application of the metadynamics technique to the modelling of crystal nucleation – a topic which has long posed challenges in structural science – and describe how detailed information can be obtained on nucleation processes for a diverse range of crystals including, ice, urea, sodium chloride and calcium carbonate.

The structural science of materials is clearly developing rapidly and the journal welcomes more submissions in this field. A full list of papers within the theme of materials and computation can be found at http://journals.iucr.org/m/services/articles_mater_comput.html.

## Figures and Tables

**Figure 1:**
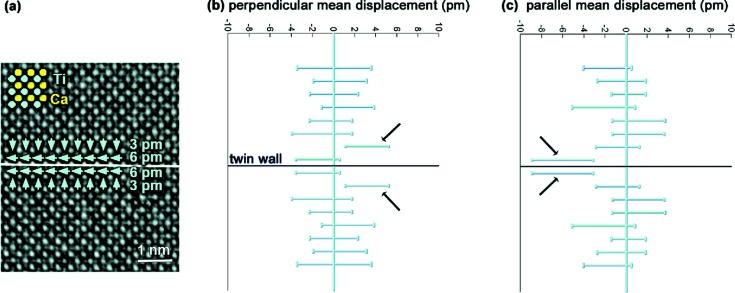
(*a*) Experimental phase image of a (110) twin boundary in orthorhombic CaTiO_3_. Mean displacements of the Ti atomic columns from the centre of the four neighbouring Ca atomic columns are indicated by green arrows. (*b*) and (*c*) Displacements of Ti atomic columns perpendicular and parallel to the twin wall, averaged along and in mirror operation with respect to the twin wall, together with their 90% confidence intervals. Reproduced from Van Aert *et al.* (2012 ▸), copyright 2011 John Wiley and Sons.
